# The Angiotensin II Type 1 Receptor C-Terminal Lys Residues Interact with Tubulin and Modulate Receptor Export Trafficking

**DOI:** 10.1371/journal.pone.0057805

**Published:** 2013-02-25

**Authors:** Xiaoping Zhang, Hong Wang, Matthew T. Duvernay, Shu Zhu, Guangyu Wu

**Affiliations:** 1 Department of Pharmacology and Experimental Therapeutics, Louisiana State University Health Sciences Center, New Orleans, Louisiana, United States of America; 2 School of Life Sciences and Technology, Tongji University, Shanghai, China; 3 Department of Pharmacology and Toxicology, Medical College of Georgia, Georgia Regents University, Augusta, Georgia, United States of America; Aix Marseille University, France

## Abstract

The physiological and pathological functions of angiotensin II are largely mediated through activating the cell surface angiotensin II type 1 receptor (AT1R). However, the molecular mechanisms underlying the transport of newly synthesized AT1R from the endoplasmic reticulum (ER) to the cell surface remain poorly defined. Here we demonstrated that the C-terminus (CT) of AT1R directly and strongly bound to tubulin and the binding domains were mapped to two consecutive Lys residues at positions 310 and 311 in the CT membrane-proximal region of AT1R and the acidic CT of tubulin, suggestive of essentially ionic interactions between AT1R and tubulin. Furthermore, mutation to disrupt tubulin binding dramatically inhibited the cell surface expression of AT1R, arrested AT1R in the ER, and attenuated AT1R-mediated signaling measured as ERK1/2 activation. These data demonstrate for the first time that specific Lys residues in the CT juxtamembrane region regulate the processing of AT1R through interacting with tubulin. These data also suggest an important role of the microtubule network in the cell surface transport of AT1R.

## Introduction

Angiotensin II (Ang II), an octapeptide hormone, modulates the physiological function of virtually all organs and plays an important role in the development of a number of human diseases such as diabetes, hypertension, myocardial infarction, congestive heart failure, and stroke. The function of Ang II is mediated through activating its cell surface receptors. There are two major subtypes of Ang II receptors, the Ang II type 1 receptor (AT1R) and the Ang II type 2 receptor (AT2R), and both receptor subtypes belong to the superfamily of seven transmembrane G protein-coupled receptors (GPCRs). It has been well documented that AT1R mediates most of the known physiological functions of Ang II. AT1R couples to the Gq protein to stimulate phospholipase C, leading to the formation of intracellular inositol 1, 4, 5-triphosphate, the release of calcium from intracellular stores and activation of mitogen-activated protein kinases (MAPK) [1]–[4].

Similar to many other GPCRs, the proper function of AT1R relies on the dynamic and highly regulated intracellular trafficking of the receptors, including anterograde export of newly synthesized receptors from the endoplasmic reticulum (ER) to the cell surface, Ang II-evoked internalization of the cell surface receptor, recycling of the internalized receptors from endosomes back to the cell surface, and targeting of the receptor to the degradation pathways. Over the past decades, the internalization, recycling and degradation of AT1R have been substantially investigated. In contrast, how AT1R transports to the cell surface remain largely unknown. A number of studies have shown that the cell surface targeting of nascent AT1R is regulated by several regulatory proteins [5]–[10] and specific motifs in the different locations within the receptor [7], [11]–[14].

Microtubules are an integral and highly dynamic component of the cytoskeletal network and are formed by polymerization of the α- and β-tubulin dimers. Both α- and β-tubulin contain the acidic EExEEY/F motif in the flexible CT. This motif coats the outer surface of microtubules and regulates microtubule interaction with many proteins (such as motor proteins) and microtubule dynamics [15], [16]. It is well known that one of the major functions of the microtubule network is to provide platforms on which many intracellular trafficking processes between organelles occur, including export from the ER and ER-to-Golgi transport of newly synthesized cargos [17]–[21]. Several studies have shown that the microtubule network coordinates endocytic transport and polarized targeting of GPCRs [22]–[28]. It has also been shown that metabotropic glutamate receptors are able to interact with tubulin [29], [30]. We have recently identified that three specific positively charged Arg residues in the CT helix 8 form the RxxxRxxxxR motif and control α_2B_-AR interaction with tubulin [31]. Here we determined the role of Lys residues located in the CT membrane-proximal region in the interaction with tubulin and cell surface transport of AT1R. We found that Lys residues, particularly the di-Lys motif Lys310/Lys311, in the CT strongly interacted with tubulin and the interaction modulated AT1R export from the ER to the cell surface. These data demonstrate for the first time that Lys residues in the CT juxtamembrane region regulate the cell surface targeting of AT1R through directly interacting with tubulin. These data also suggest that highly conserved basic residues in the CTs of GPCRs may function as a common code to direct receptor contact with the microtubule network to coordinate their ER-to-cell surface movement.

## Materials and Methods

### Materials

α-tubulin antibodies (DM1A) were obtained from Sigma-Aldrich (St. Louis, MO). High affinity anti-HA-fluorescein (3F10) was from Roche Molecular Biochemicals (Mannheim, Germany). The ER marker pDsRed2-ER was from BD Biosciences (Palo Alto, LA). Purified bovine tubulin was purchased from Cytoskeleton Inc. (Denver, CO). Tubulin lacking the acidic CT (tubulin S) prepared by limited proteolysis of rat tubulin with subtilisin as described [32], [33] was kindly provided by Dr. Dan L. Sackett (Eunice Kennedy Shriver National Institute of Child Health and Human Development, National Institutes of Health). Antibodies against ERK1/2 and phospho-ERK1/2 were from Cell Signaling Technology (Beverly, MA). Prolong antifade reagent with DAPI was obtained from Invitrogen Life Technologies (Carlsbad, CA). Rat brains were purchased from Pel-Freez Biologicals (Rogers, Arkansas). All other materials were obtained as described elsewhere [13], [31].

### Plasmid Constructions

AT1R tagged with green fluorescent protein (GFP) at its CT (AT1R-GFP) and AT1R tagged with three hemagglutinin (HA) at its N-terminus (HA-AT1R) were generated as described previously [5], [13]. The GFP and HA epitopes have been used to label GPCRs including AT1R, resulting in receptors with similar characteristics to the wild-type receptors [8], [11], [13], [34]. Glutathione S-transferase (GST) fusion protein constructs coding the CTs of α_2B_-AR, AT1R and β_2_-AR were generated in the pGEX-4T-1 vector as described previously [31], [35], [36]. All mutants were made with the Quick Change site-directed mutagenesis kit. The sequence of each construct was confirmed by restriction mapping and nucleotide sequence analysis.

### Cell Culture and Transient Transfection

HEK293 cells were cultured in Dulbecco’s modified Eagle’s medium (DMEM) with 10% fetal bovine serum (FBS), 100 units/ml penicillin, and 100 units/ml streptomycin. Transient transfection of the cells was carried out using Lipofectamine 2000 reagent (Invitrogen, Carlsbad, CA) as described previously [5]. Transfection efficiency was estimated to be greater than 80% based on the GFP fluorescence.

### GST Fusion Protein Pulldown Assays

The GST-fusion proteins were expressed in bacteria and purified using a glutathione affinity matrix as described previously [31], [35], [36]. Immobilized fusion proteins were either used immediately or stored at 4°C for no longer than 3days. Each batch of fusion protein used in experiments was first analyzed by staining with Coomassie Brilliant Blue R-250 following SDS-PAGE. Tubulin purified from bovine brain was reconstituted in general tubulin buffer (G-PEM: 80 mM PIPES pH6.9, 2 mM MgCl_2_ **,** 0.5 mM EGTA, 50 mM GTP). Two µl of GST fusion proteins bound to glutathione-Sepharose beads were incubated with 1 µg of purified tubulin or tubulin S in G-PEM plus 2% NP-40 and 100 mM NaCl for 1 h at room temperature. For interaction with tubulin from brain extracts, rat brains were homogenized in buffer containing 50 mM Tris-HCl, pH 7.4, 5 mM EDTA, 5 mM EGTA, 9 mM KCl, 2.5 mM MaCl_2_, 1% Triton X-100. After centrifugation at 100,000 × g for 45 min at 4°C, 100 µg of the supernatant were incubated with 10 µl of GST fusion proteins bound to glutathione-Sepharose beads in homogenization buffer plus 100 mM NaCl. The resin was then washed three times with binding buffer. The bound proteins were solubilized in 20 µl of 2 × SDS-gel loading buffer and separated by 10% SDS-PAGE. The bound tubulin was detected by Western blotting using α-tubulin specific antibodies.

### Flow Cytometry

For measurement of AT1R expression at the cell surface, HEK293 cells were cultured on 6-well dishes and transfected with 1 µg of HA-AT1R for 24–36 hrs. The cells were collected, suspended in phosphate-buffered saline (PBS) containing 1% FBS at a density of 1×10^7 ^cells/ml and incubated with high affinity anti-HA-fluorescein (3F10) at a final concentration of 2 µg/ml for 30 min at 4°C. For measurement of total AT1R expression, HEK293 cells were transiently transfected with AT1R-GFP. The cells were collected, washed twice with PBS and re-suspended. The fluorescence was measured on a flow cytometer (BD Biosciences FASCalibur) as described previously [13], [37].

### Measurement of ERK1/2 Activation

HEK293 cells were cultured in 6-well dishes and transfected with 0.5 µg of AT1R or its mutant. At 6-8 h after transfection, the cells were split into 6-well dishes and cultured for additional 36 h. The cells were starved for at least 3 h and then stimulated with 1 µM Ang II for 2 min at 37°C. Stimulation was terminated by addition of 1×SDS-gel loading buffer. After solubilizing the cells, 20 µl of total cell lysates was separated by 12% SDS-PAGE. ERK1/2 activation was determined by measuring the levels of phosphorylation of ERK1/2 with phospho-specific ERK1/2 antibodies by immunoblotting [5].

### Fluorescence Microscopy

Subcellular distribution of AT1R and its co-localization with the ER marker DsRed2-ER was analyzed by fluorescence microscopy as described previously [38]. Briefly, HEK293 cells were grown on coverslips pre-coated with poly-L-lysine in 6-well plates and transfected with 100 ng of GFP-tagged AT1R for 36 to 48 hrs. The cells were fixed with 4% paraformaldehyde-4% sucrose mixture in PBS for 15 min. The coverslips were mounted with prolong antifade reagent and fluorescence was detected with a Leica DMRA2 epifluorescent microscope. Images were deconvolved using SlideBook software and the nearest neighbor deconvolution algorithm (Intelligent Imaging Innovations, Denver, CO). For visualization of AT1R tagged with HA, HEK293 cells were permeabilized with PBS containing 0.2% Triton X-100 for 5 min and blocked with 5% normal donkey serum for 1 h. The cells were then incubated with anti-HA antibodies for 1 h. After washing with PBS (3×5 min), the cells were incubated with Alexa Fluor 594-labeled secondary antibody (1:2000 dilution) for 1 h at room temperature. For co-localization studies, HEK293 cells were transfected with AT1R-GFP together with pDsRed2-ER. The colocalization of the receptor with the ER marker DsRed2-ER was determined by Pearson’s coefficient using the ImageJ JaCoP plug-in as described [39].

### Statistical Analysis

Differences were evaluated using Student's *t* test, and *p* < 0.05 was considered as statistically significant. Data are expressed as the mean ± S.E.

## Results

### Interaction of the CT of AT1R, but not β_2_-AR, with Tubulin

**Figure 1:**
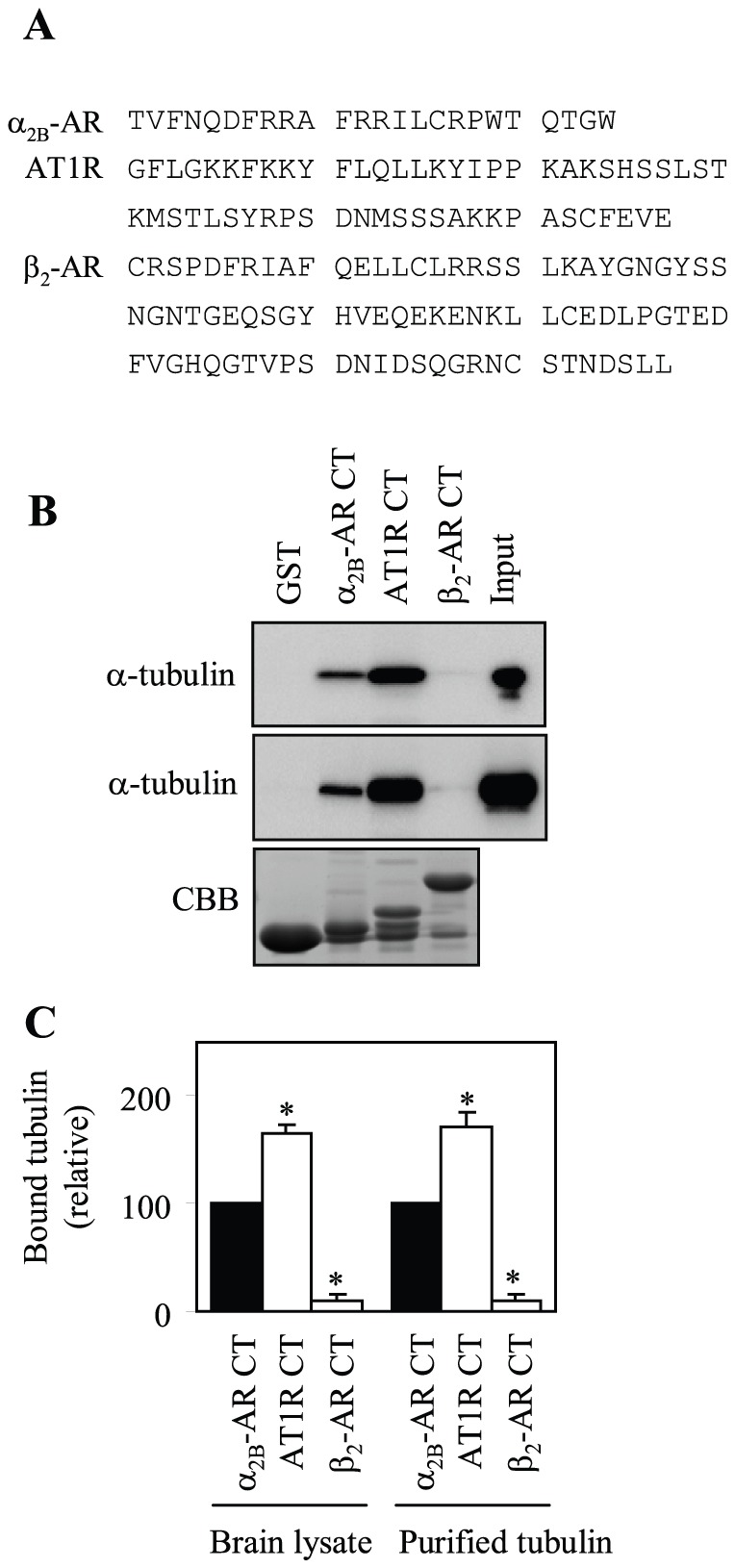
Interaction of the CTs of α_2B_-AR, AT1R and β_2_-AR with tubulin. (A) Sequence of the CTs of α_2B_-AR, AT1R and β_2_-AR. (B) Interaction of the CTs with tubulin from rat brain extracts and purified tubulin. GST and GST fusion proteins encoding the CTs of α_2B_-AR, AT1R and β_2_-AR were incubated with 100 µg of rat brain extracts (top panel) or 1 µg of purified tubulin (middle panel) for 1 h at room temperature as described in “Materials and Methods”. The amount of tubulin bound to GST fusion proteins was analyzed by Western blotting usingα-tubulin antibodies. Bottom panel: Coomassie Brilliant Blue (CBB) staining of purified fusion proteins after SDS-PAGE. (C) Quantitative data expressed as percentages of tubulin interacting with the α_2B_-AR CT and presented as the mean ± S.E. of three separate experiments. *, p < 0.05 versus the α_2B_-AR CT.

To determine if the AT1R CT is able to interact with tubulin, the α_2B_-AR CT (which has multi positively charged residues and has been shown to interact with tubulin [31]) was used as a positive control and GST alone and the β_2_-AR CT (which does not have any highly positively charged regions) were used as possible negative controls (Fig. 1A). The CTs of AT1R, β_2_-AR and α_2B_-AR were generated as GST fusion proteins and their interaction with brain extracts and purified tubulin was determined by GST fusion protein pulldown assay. The AT1R CT interacted with tubulin from brain extracts and purified tubulin (Fig. 1B) and the amount of tubulin bound to the AT1R CT was significantly higher than that bound to the α_2B_-AR CT (Fig. 1C). In contrast, GST and the GST fusion proteins encoding the β_2_-AR CT did not bind to tubulin (Fig. 1B and 1C). These data demonstrate that the CT of AT1R, but not β_2_-AR, is able to directly and strongly associate with tubulin.

### Identification of the Motifs Mediating the Interaction between AT1R and Tubulin

**Figure 2:**
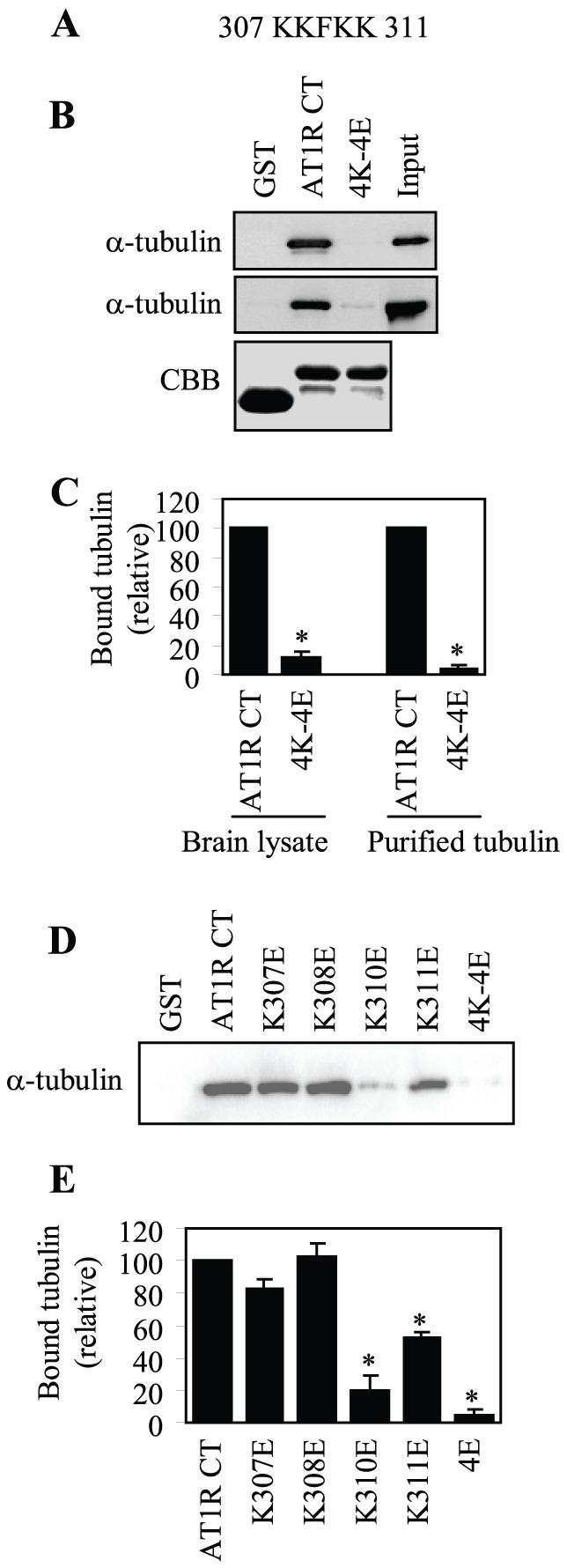
Identification of essential residues in the AT1R CT for tubulin interaction. (A) Sequence of the AT1R CT containing four Lys residues at positions 307, 308, 310 and 311. (B) Effect of mutating the four Lys residues to Glu on the AT1R CT interaction with tubulin. GST and GST fusion proteins encoding the AT1R CT or its mutant in which the four Lys residues were mutated to Glu (4K-4E) were incubated with rat brain cytosolic extracts (top panel) or purified tubulin (middle panel) as described in the legend of Fig. 1B. Bottom panels: Coomassie Brilliant Blue (CBB) staining of purified GST fusion proteins after SDS-PAGE. (C) Quantitative data of (B). (D) Effect of mutating individual Lys residue on the AT1R CT interaction with purified tubulin. GST and GST fusion proteins encoding the AT1R CT or its mutants in which Lys residues at positions 307, 308, 310 and 311 were individually mutated to Glu were incubated with purified tubulin as described in the legend of Fig. 1B. E, Quantitative data of (D). In (C) and (E), quantitative data are expressed as percentages of tubulin interacting with the AT1R CT and presented as the mean ± S.E. of at least three individual experiments. *, p < 0.05 versus the AT1R CT.

To identify specific residues in the AT1R CT responsible for interaction with tubulin, we focused on four Lys residues at positions 307, 308, 310 and 311 in the membrane-proximal region (Fig. 2A). The four Lys residues were first mutated to Glu together (4K-4E), which will presumably preserve the amphipathic character of the helix 8. The effect of mutation on the AT1R CT interaction with tubulin was determined in the GST fusion protein pulldown assay. Mutation of all four Lys residues almost completely blocked the AT1R CT interaction with both tubulin from brain extracts and purified tubulin (Fig. 2B and 2C).

We then determined the effect of mutating individual Lys residue in the 307KKxKK311 motif on the AT1R CT interaction with tubulin. Mutation of Lys310 to Glu profoundly inhibited the AT1R CT interaction with tubulin and mutation of Lys311 to Glu also significantly attenuated the interaction (Fig. 2D and 2E). In contrast, mutation of Lys307 and Lys308 did not produce clear inhibitory effects on the AT1R CT interaction with tubulin (Fig. 2D and 2E). These data indicate that different Lys residues in the 307KKxKK311 motif differently contribute to the AT1R interaction with tubulin and the AT1R CT interaction with tubulin is largely mediated through the di-Lys motif Lys310/Lys311.

**Figure 3:**
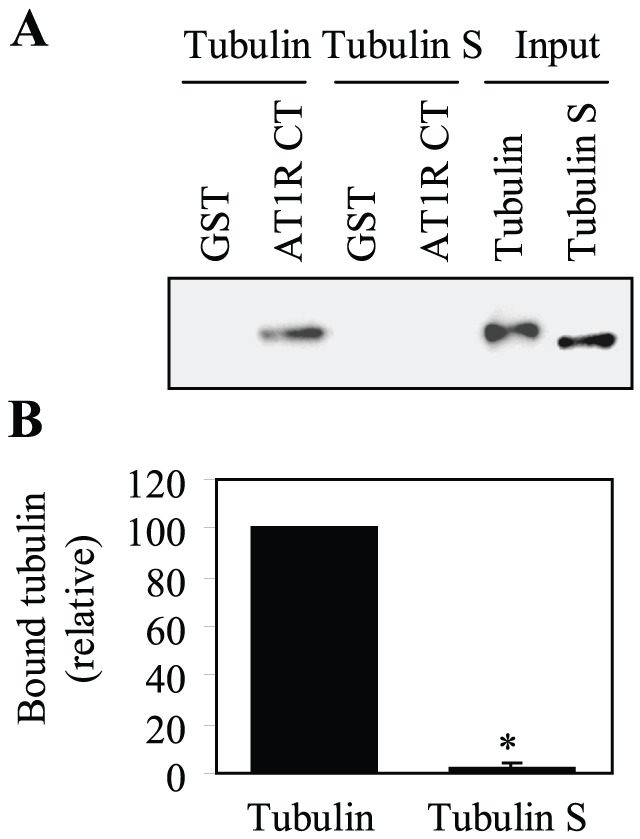
Effect of removing the CT of tubulin on its interaction with the AT1R CT. (A) GST and GST fusion proteins encoding the AT1R CT were incubated with 1 µg of purified tubulin or tubulin S in which the negatively charged CT was cleaved. The bound tubulin and tubulin S were determined by Western blotting usingα-tubulin antibodies. (B) Quantitative data expressed as percentages of tubulin interacting with the AT1R CT and presented as the mean ± S.E. of three separate experiments. *, p < 0.05 versus tubulin.

To identify the AT1R-binding site on tubulin, we compared the AT1R CT interaction with tubulin and tubulin S, a truncated form of tubulin in which the negatively charged CT was cleaved [32], [33]. GST fusion protein pulldown assay showed that the AT1R CT did not interact with tubulin S (Fig. 3A and 3B). These data suggest that the interaction between AT1R and tubulin is essentially ionic.

### Role of Tubulin Interaction in AT1R Transport from the ER to the Cell Surface

**Figure 4:**
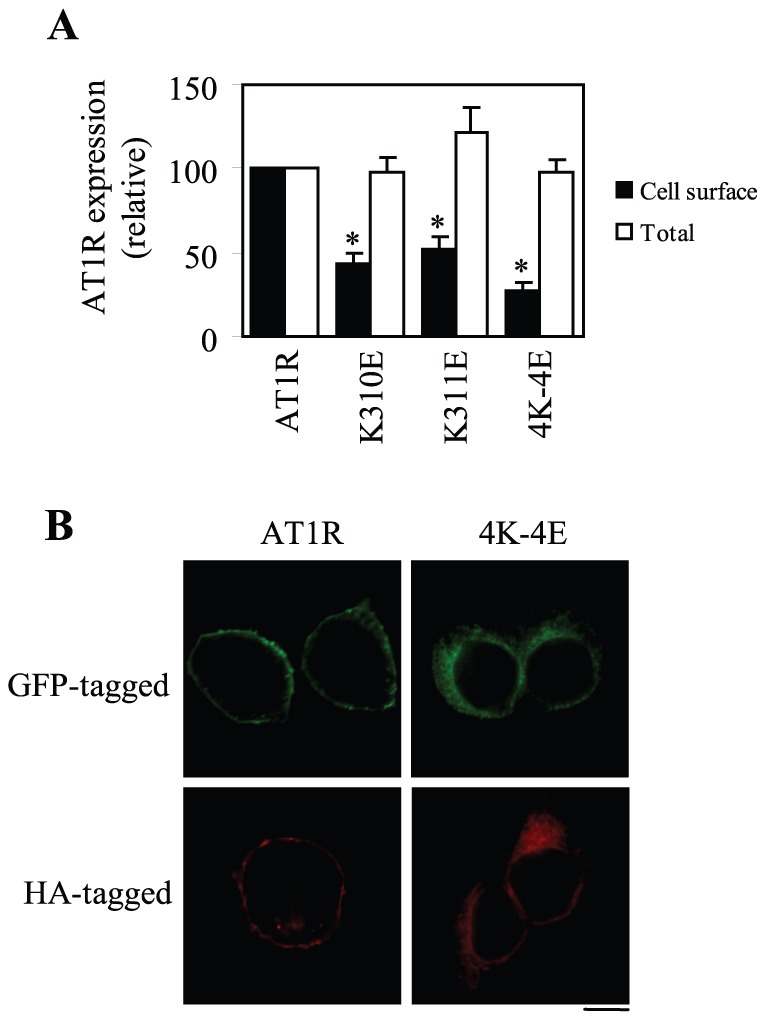
Effect of mutating Lys residues on the cell surface and total expression of AT1R. (A) AT1R and its mutants K310E, K311E and 4K–4E were either tagged with three HA at their N-termini or with GFP at their C-termini. HEK293 cells were transfected with HA-tagged receptors and the cell surface expression of the receptors was measured by flow cytometry following staining with HA antibodies in non-permeablized cells as described in “Materials and Methods.” In separate experiments, HEK293 cells were transfected with GFP-tagged receptors and total receptor expression was determined by flow cytometry detecting the GFP signal. The data shown are percentages of the mean value obtained from cells transfected with wild-type AT1R and are presented as the mean ± S.E. of at least six experiments. *, p < 0.05 versus AT1R. (B) Effect of mutating the tubulin-binding site on the subcellular distribution of AT1R. HEK293 cells cultured on coverslips were transfected with AT1R or its mutant 4K–4E tagged with GFP (upper panel) or HA (lower panel). The subcellular distribution of GFP-AT1R was revealed by fluorescence microscopy to directly detecting GFP, whereas HA-AT1R was detected following staining with anti-HA antibodies. Scale bar, 10 µm.

To study the function of AT1R interaction with tubulin, we first determined the effect of mutating the tubulin-binding motif on the cell surface expression of AT1R. AT1R and its mutants K310E, K311E and 4K–4E which have reduced ability to interact with tubulin were tagged with three HA at their N-termini and their cell surface expression was measured by flow cytometry following staining with anti-HA antibodies in non-permeabilized cells. Meanwhile, AT1R and its mutants were tagged with GFP at their CTs and their total expression was determined by measuring GFP signal by flow cytometry. In parallel with the interaction with tubulin, the cell surface expression of the mutants 4K–4E, K310E and K311E was significantly attenuated by 73, 57 and 48%, respectively (Fig. 4A). In contrast, total expression of these mutants was comparable to that of wild-type AT1R (Fig. 4A). These data suggest that AT1R interaction with tubulin modulates its cell surface expression.

To further confirm the effects of mutating the tubulin-binding site on AT1R transport as measured by flow cytometry, the subcellular distribution of AT1R and its 4K–4E mutant which has profound defects in the cell surface transport was visualized by fluorescence microscopy. As expected, wild-type AT1R tagged with either GFP or HA robustly expressed at the cell surface, whereas the mutant 4K–4E was strongly expressed in the perinuclear region (Fig. 4B).

### Role of Tubulin Interaction in AT1R Exit from the ER

**Figure 5:**
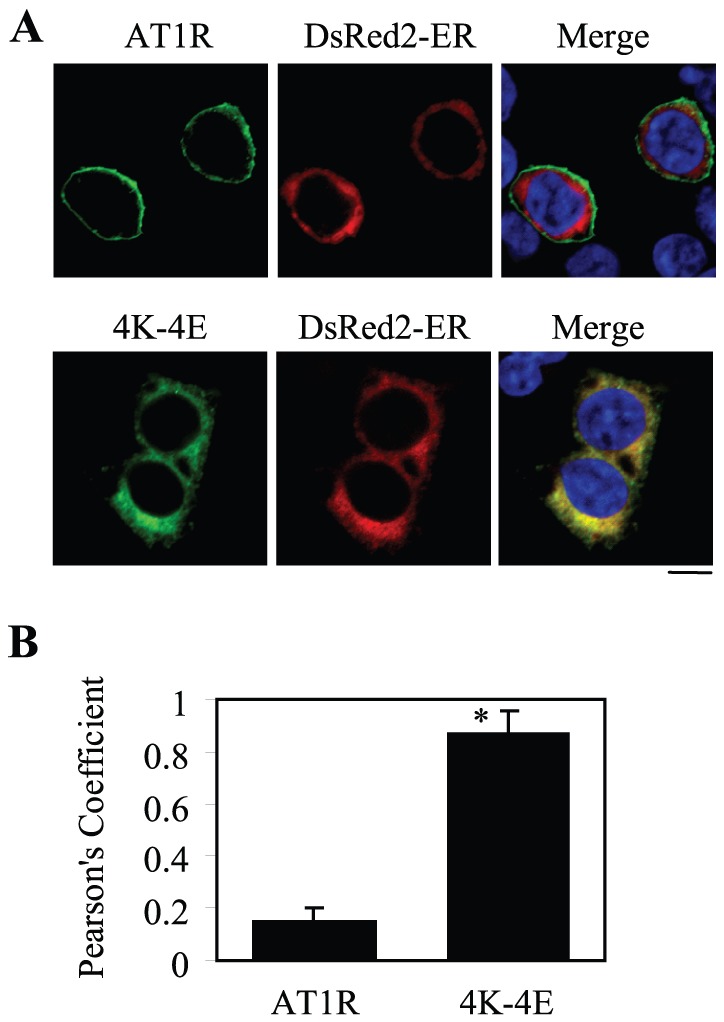
Effect of mutating the tubulin-binding site on AT1R export from the ER. (A) Co-localization of AT1R and its mutant 4K–4E with the ER marker DsRed2-ER. HEK293 cells cultured on coverslips were transfected with GFP-tagged AT1R or its mutant 4K–4E together with pDsRed2-ER. Co-localization of AT1R and 4K–4E with DsRed2-ER was revealed by fluorescence microscopy. Green, GFP-tagged AT1R; red, the ER marker DsRedER-2; yellow, co-localization of AT1R with the ER marker; blue, DNA staining by 4,6-diamidino-2-phenylindole (nuclei). The data shown are representative images of at least four independent experiments. (B) Quantification of Pearson’s coefficient between the receptors and the ER marker. The data are presented as the mean ± S.E. of 60 cells from four different experiments. *, *p* < 0.05 *versus* wild-type AT1R. Scale bar, 10 µm.

To define the intracellular compartment in which the mutant 4K–4E was accumulated, GFP-tagged AT1R and its mutant 4K–4E were co-localized with different intracellular markers. The mutant 4K–4E was extensively co-localized with the ER marker DsRed2-ER (Fig. 5A), but not the Golgi marker GM130 (data not shown). In contrast, wild-type AT1R did not clearly co-localize with DsRed2-ER (Fig. 5A) and the Golgi marker GM130 (data not shown). To quantify the colocalization of AT1R with the ER marker, Pearson’s coefficient was determined. Pearson’s coefficient of the mutant 4K–4E was significantly higher than that of wild-type AT1R (Fig. 5B). These data suggest that tubulin interaction may play an important role in regulating AT1R export from the ER.

### Effect of Mutating the Four Lys Residues on AT1R Signaling

**Figure 6:**
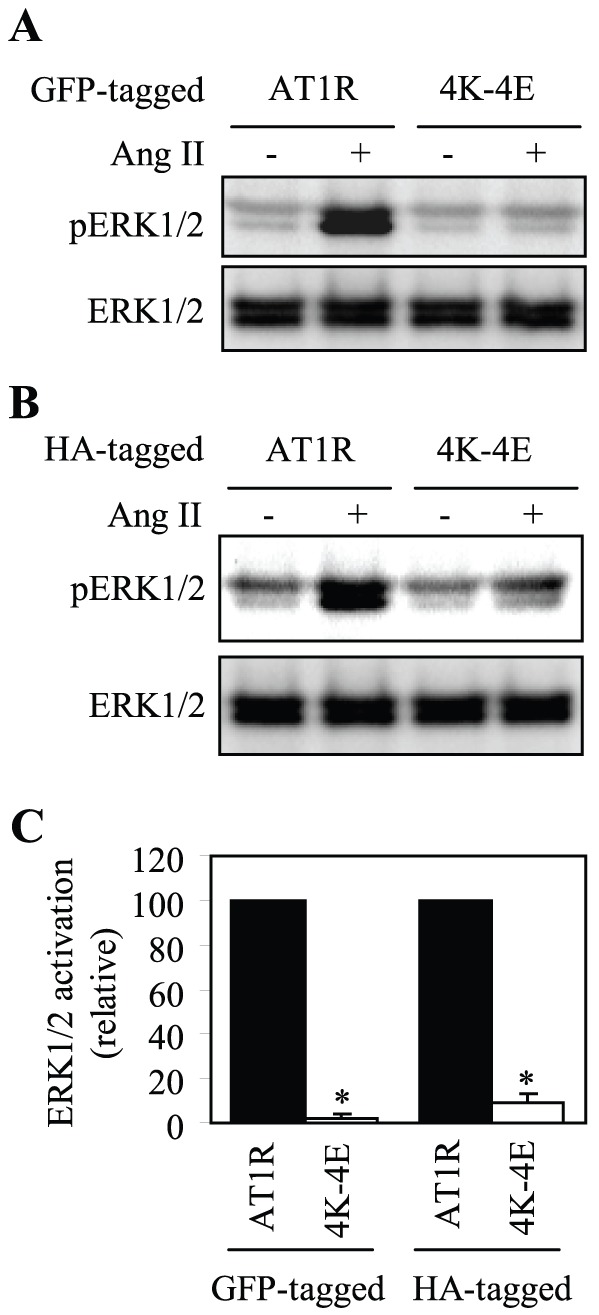
Effects of mutating the tubulin-binding site on AT1R-mediated activation of ERK1/2. (A) and (B) HEK293 cells were transfected with wild-type AT1R or its mutant 4K–4E tagged with GFP at their C-termini (A) or with HA at their N-termini (B). The cells were then stimulated with Ang II at a concentration of 1 µM for 2 min. ERK1/2 activation was determined by Western blot analysis using phospho-specific ERK1/2 antibodies. *Upper panels*, representative blots of ERK1/2 activation; *Lower panels*, total ERK1/2 expression. (C) Quantitative data expressed as percentages of ERK1/2 activation obtained in cells transfected with AT1R and presented as the mean ± S.E. of four separate experiments. *, *p* < 0.05 *versus* respective AT1R.

Our preceding data have shown that mutation of the four Lys residues to Glu disrupts the AT1R CT interaction with tubuln and AT1R transport from the ER to the cell surface. We next determined if this mutation could alter AT1R function by using the Ang II-mediated activation of ERK1/2 as readout. HEK293 cells were transiently transfected with AT1R and its mutant 4K–4E tagged with either GFP at their CTs (Fig. 6A) or HA at their N-termini (Fig. 6B). The abilities of AT1R and its mutant to activate ERK1/2 in response to stimulation with Ang II were compared. In our system, the maximal activation of ERK1/2 in cells expressing wild-type AT1R was achieved after stimulation with Ang II at a concentration of 1 µM for 2 min (Fig. 6A and 6B) and higher concentrations of Ang II and/or longer stimulation reduced ERK1/2 activation which is likely due to the internalization of AT1R (data not shown). In contrast, the activation of ERK1/2 by Ang II was almost abolished in cells expressing the mutant 4K–4E (Fig. 6A and 6B). These data are consistent with the marked reduction in the cell surface expression of the mutant 4K–4E as compared with its wild-type counterpart.

## Discussion

The molecular mechanisms underlying the export trafficking of AT1R remain largely unknown. In this study, we have demonstrated that the AT1R CT bound to tubulin and the interaction is specifically mediated through positively charged Lys resides, particularly those at positions 310 and 311, in the AT1R CT and the tubulin CT. Furthermore, mutation of the tubulin-binding site to disrupt tubulin interaction significantly attenuated the cell surface transport of AT1R. These data are consistent with previous studies showing that mutation of Lys310/Lys311 and all four Lys in the sequence 307KKFKK311 markedly attenuated the cell surface expression of AT1R [14]. In parallel with the effect of the mutation on the cell surface expression of AT1R, ERK1/2 activation was dramatically inhibited in cells expressing the mutant as compared with cells expressing wild type AT1R. It is strongly likely that the signaling attenuation is due to the inability of the mutated receptors to transport to the cell surface. Nevertheless, these data provide strong evidence indicating that AT1R may directly contact with the microtubule network via ionic interactions with tubulin to coordinate its ER-to-cell surface traffic, a novel mechanism controlling AT1R export to the functional destination.

**Figure 7:**
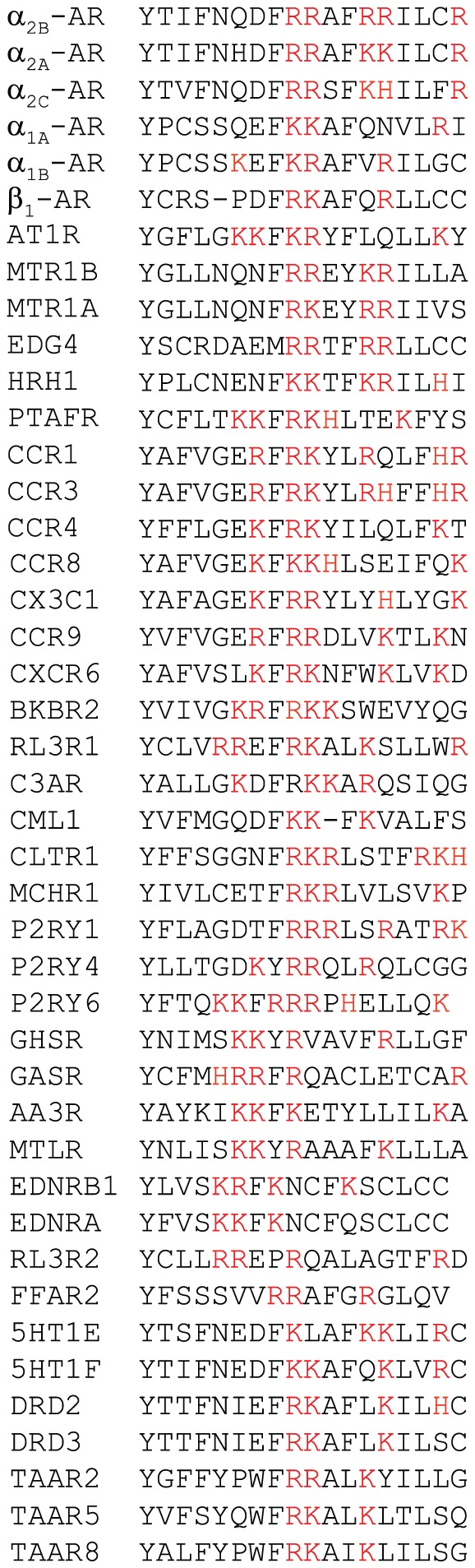
The highly conserved basic residues in the CTs of family A GPCRs. Human sequences are shown when available. (A) AR, adrenergic receptor; AT1R, angiotensin II type 1 receptor; MTR, melatonin receptor; EDG4, lysophosphatidic acid receptor; HRH1, histamine H1 receptor; CCR, C–C chemokine receptor; BKRB2, B2 bradykinin receptor; RL3R1, relaxin-3 receptor; C3AR, C3a anaphylatoxin chemotactic receptor; CML1, chemokine receptor-like 1 receptor; CLTR, cysteinyl leukotriene receptor; MCHR, melanin-concentrating hormone receptor; P2RY, purinergic receptor; GHSR, growth hormone secretagogue receptor; GASR, gastrin/cholecystokinin receptor; AA3R, adenosine A3 receptor; MTLR, motilin receptor; EDNR, endothelin receptor; RL3R, relaxin-3 receptor; FFAR, free fatty acid receptor; 5HT, serotonin receptor; DRD, dopamine receptor; TAAR, trace amine-associated receptor. The first residue Tyr in the alignment represents the end of the 7^th^ transmembrane domain characterized by the NPxxY motif.

Importantly, we have previously reported that α_2B_-AR uses the positively charged Arg residues in the CT to directly interact with the negatively charged CT of tubulin [31]. The positively charged residues are highly conserved in the CT membrane-proximal portions and appear to cluster on helix 8 in an amphipathic pattern family A GPCRs (Fig. 7). Most of these charged residues are spaced apart by one (e.g. AT1R) or two residues (e.g. α_2_-ARs). Therefore, the positively charged residue clusters in the CT juxtamembrane regions of GPCRs may function as a common signal directing GPCR traffic along the microtubule network.

Our studies have revealed that the positive charges are an important determinant for the function of the CT basic residues in mediating GPCR interaction with tubulin and different GPCRs may use distinct positively charged motifs to interact with tubulin. Specifically, our previous studies have demonstrated that mutation of Arg residues at positions 437, 410 and 446 produced more potent inhibitory effects on α_2B_-AR interaction with tubulin than mutation of Arg residues at positions 438 and 411. Our current studies have shown that mutation of Lys residues at positions 310 and 311 markedly inhibited AT1R interaction with tubulin, whereas mutation of Lys residues at positions 307 and 308 did not have inhibitory effects. These data have revealed two distinct positively charged tubulin-binding motifs in different GPCRs: a triple Arg motif 437RxxxRxxxxR446 in α_2B_-AR and a double Lys motif 310KK311 in AT1R. These data also suggest that the positive charge property of the basic residue clusters is not the only factor that dictates GPCR interaction with tubulin. The function of the basic residues in the membrane-proximal CT in mediating GPCR interaction with tubulin may also be determined by their structural features, such as the locations of individual basic residue in the cluster and the positions of the cluster relative to the 7^th^ transmembrane domain. It is also interesting to note that mutation of Lys310 caused more dramatic inhibitory effect on AT1R interaction with tubulin than mutation of Lys311, suggesting that different basic residues in the same tubulin-binding motif differentially contribute to the interaction with tubulin. Nevertheless, the data presented in the current manuscript, together with our previous studies, strongly indicate that, similar to many microtubule plus-end tracking proteins including microtubule associated proteins [15], [16], GPCR interaction with tubulin is mediated through very specific positively charged residues.

In contrast to the well-defined regulators involved in endocytosis, recycling and degradation of GPCRs, players that coordinate the export trafficking of newly synthesized GPCRs from the ER to the cell surface have just begun to be revealed [5], [8], [11], [13], [35]–[38], [40]–[48]. For AT1R, we have demonstrated that its transport to the cell surface is regulated by several Ras-like small GTPases, including Rab1, Rab2, Rab6, Sar1, and ARF1 [5]–[9]. We have also demonstrated that AT1R export from the ER is controlled by a number of specific motifs in the different locations within the receptor, such as a single Leu residue in the first intracellular loop and the F(x)_6_LL and ExE motifs in the CT (Fig. 7B) [7], [11]–[13]. The F residue in the F(x)_6_LL motif is likely involved in the regulation of AT1R export from the ER via influencing correct receptor folding, whereas double Leu residues in the F(x)_6_LL motif may alter receptor cell surface transport from the ER and the Golgi [11], [35], [38]. The di-acidic ExE motif in the CT may be involved in the regulation of receptor targeting onto the COPII vesicles which mediate protein transport exclusively from the ER [37]. As demonstrated in this study, the code-directed specific interaction of AT1R with the microtubule network provides additional important checkpoints for modulating the transport of nascent AT1R to the functional destination, which will ultimately control the physiological and pathological functions of the receptor. Since the trafficking of GPCRs plays a crucial role in regulating receptor functionality, and indeed, dysfunction of GPCRs in general or AT1R in particular that are caused by defective cell surface trafficking is clearly associated with the development of a number of human diseases [4], [49]–[51], to thoroughly understand the mechanism underlying export trafficking of GPCRs will provide a foundation for the development of novel therapeutic strategies to manipulate the amount of the functional receptors at the cell surface.
